# Reactive oxygen species metabolism-based prediction model and drug for patients with recurrent glioblastoma

**DOI:** 10.18632/aging.102506

**Published:** 2019-12-04

**Authors:** Nian Tan, Jianwei Liu, Ping Li, Zhaoying Sun, Jianming Pan, Wei Zhao

**Affiliations:** 1 Department of Human Anatomy, College of Integrated Traditional Chinese and Western Medicine, Tianjin University of Traditional Chinese Medicine, Tianjin, P. R. China

**Keywords:** glioblastoma, tumor recurrence, reactive oxygen species, prognosis, Chinese herb

## Abstract

Background: Tumor recurrence is the main cause of poor prognosis of GBM. Finding the characteristics of recurrent GBM that provide early warning of tumor recurrence can provide guidance for the clinical treatment of recurrent GBM.

Results: Reactive oxygen species (ROS) biosynthetic processes was significantly elevated in recurrent GBM. The recurrent risk score based on the ROS biosynthetic process was closely related to tumor purity and tumor immune functions. The quantitative risk assessment system could be used to predict the recurrence time of GBM. Gallic acid, a compound with high anti-oxidation activity and low cytotoxicity, was screened as a potential chemotherapy sensitizer for recurrent GBM.

Conclusion: The quantitative risk assessment system based on ROS biosynthetic process could be used for early warning of GBM recurrence. Combination of low-dose gallic acid and temozolomide could improve therapeutic outcomes in recurrent GBM.

Methods: A total of 663 primary and recurrent GBM samples with clinical and microarray data were included in this study. GSVA, LASSO-COX, and Kaplan-Meier survive curve were performed to construct and verify a quantitative risk assessment system for GBM recurrence prediction. An antioxidant capacity test and cell viability test were used to discover potential drugs for recurrent GBM.

## INTRODUCTION

Glioblastoma (GBM) is the most common and highly lethal malignancy of the central nervous system [1]. Standard treatment for GBM includes total tumor resection and postoperative adjuvant radiotherapy and chemotherapy [1, 2]. In clinical practice, we have found that even after aggressive treatment, the recurrence of GBM is almost inevitable. Temozolomide (TMZ), as the first-line chemotherapeutic drug for gliomas, is widely used in patients with recurrent GBM, although most tumors are insensitive to this drug. Lack of effective treatment for recurrent GBM is one of the main causes of poor prognosis [3, 4]. Therefore, a mechanistic understanding of early warning signals that predict recurrence may help to prolong the survival of GBM patients.

In recent years, exploration of pathogenesis and development of novel treatments of recurrent GBM has become an increasingly active area of research [5]. Previous studies have identified many markers associated with tumor recurrence, which were used to develop corresponding targeted therapies [6–8]. However, due to the selective and variable expression of most molecular markers, molecular targeted therapy cannot benefit most GBM patients [9, 10]. Therefore, finding the biological functions on which tumor recurrence depends and applying the corresponding targeted drugs may provide benefits to a wider range of patients.

Prediction of tumor recurrence is an important area of GBM research, and many GBM recurrence prediction models have been established over the past decades [11–13]. Unfortunately, most prediction models cannot be clinically applied due to poor accuracy or complexity. Currently, the monitoring of GBM recurrence in the clinic is still by regular MRI review and symptom changes. However, this traditional method is inefficient and costly. Therefore, it is imperative to establish a simple, easy-to-detect and highly accurate prediction model for early prediction of tumor recurrence in the clinic.

Natural medicines, especially Chinese herbal medicine, are widely used in tumor therapy [14]. The application of arsenic trioxide, an extract of arsenic, significantly improved the clinical cure rate of acute promyelocytic leukemia patients [15]. Many other herbs have been used as immunomodulators or chemoradiotherapy sensitizers in adjuvant therapy for many tumors, such as lung cancer [16], melanoma [17] and glioma [18]. However, there is still no research on the application of herbs to recurrent GBM, even though it is not sensitive to traditional therapy.

In our study, functional analysis was applied to screen for specific elevated biological functions in recurrent GBM. It was found that ROS biosynthetic processes were steadily elevated in recurrent GBM. Subsequently, the recurrent risk score was constructed by dimensionality analysis of the ROS biosynthetic process-related genes. Functional analysis found that ROS biosynthetic process was closely related to tumor immune checkpoints in GBM. Based on the recurrent risk score and other recurrence-related risk factors, a quantitative risk assessment (QRA) system for GBM recurrence time prediction was constructed. Finally, we found that among 12 kinds of Chinese herbal extracts with antioxidant effects, gallic acid showed the strongest ROS scavenging ability. The combination of low-dose gallic acid and TMZ could significantly improve the chemosensitivity of TMZ in TMZ-resistant glioma cell line. This prediction model and chemosensitizer could be of potential value in the clinic.

## RESULTS

### Reactive oxygen species biosynthetic processes were highly enriched in recurrent GBM

**Figure 1:**
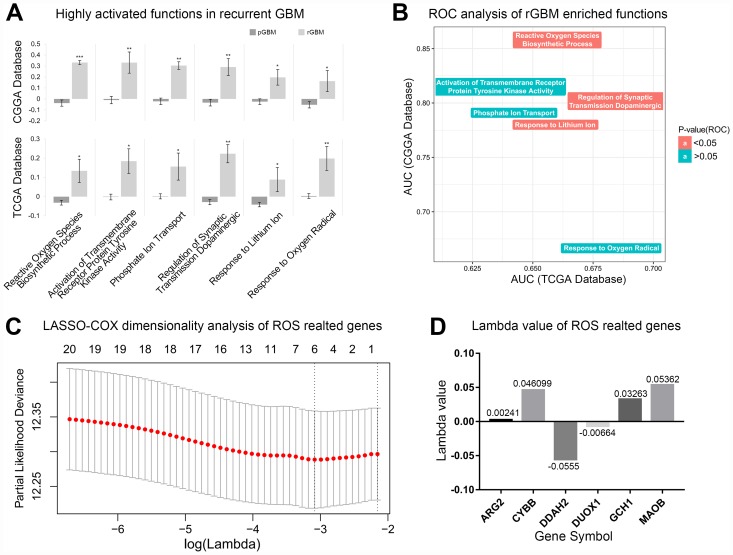
**Reactive oxygen species biosynthetic processes were significantly elevated in recurrent GBM.** (**A**) Significantly elevated biological functions in recurrent GBM. pGBM represents primary GBM and rGBM represents recurrent GBM. ^*^p<0.05, ^**^p<0.01, ^***^p<0.001. (**B**) AUC values of recurrent GBM enriched biological functions in TCGA and CGGA databases. (**C**) The most representative genes were obtained by LASSO-COX dimensionality analysis of ROS related genes. (**D**) The lambda value of 6 most representative genes.

In order to find highly activated functions in recurrent gliomas, a GSVA analysis of 4,436 biological processes of primary and recurrent GBM were performed. Of these, only 6 biological processes were significantly increased in recurrent GBM in samples in the CGGA and TCGA databases (Figure 1A). Next, the specificity of the above biological process enrichment in recurrent GBM was revealed by ROC analysis. Three specific enriched processes were regarded as candidates for further analysis (Figure 1B). In order to make the results more reliable, the above results were verified against primary/recurrent matched GBM samples. Finally, only ROS biosynthetic process showed high stability and specific enrichment in recurrent GBM (Supplementary Figure 1). Among 23 genes involved in ROS biosynthetic process, 6 most important genes were screened by LASSO-COX dimensional analysis to determine feasibility of clinical translation (Figure 1C). According to the expression of the above 6 genes and their corresponding lambda values (Figure 1D), the recurrent risk score for each patient could be calculated. Details in Methods section.

### The landscape of recurrent risk scores and clinicopathologic characteristics in GBM

**Figure 2:**
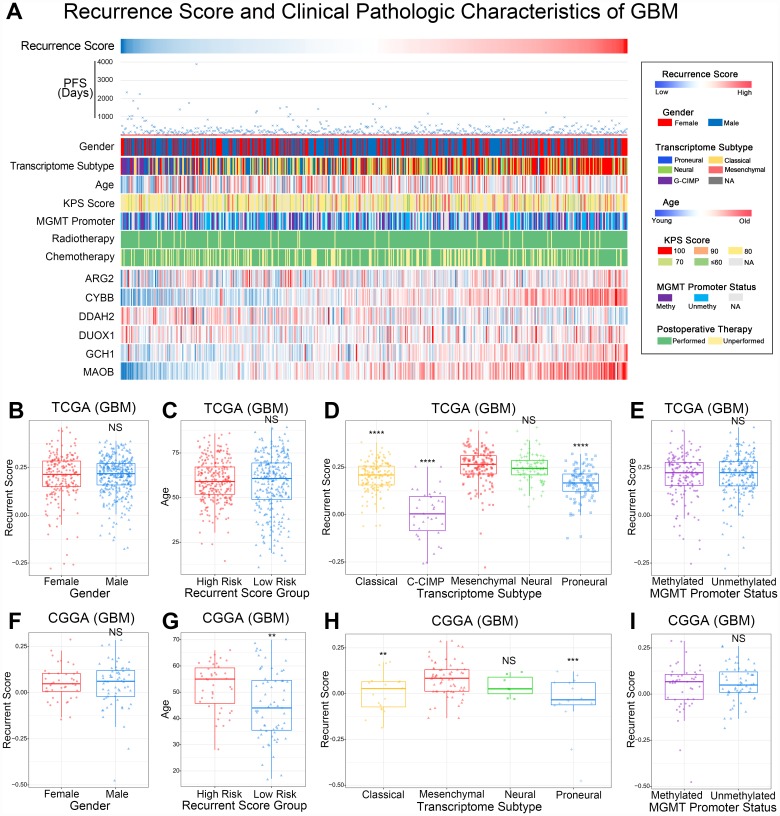
**The relationship between recurrence score and clinical pathologic characteristics of GBM.** (**A**) The heatmap presented PFS days, clinical pathology, and 6 representative genes of ROS for each GBM patient in ascending order of the recurrence score. PFS days, transcriptome subtype, and 6 ROS related genes showed asymmetry distribution. (**B**, **F**) The recurrence score showed no significant difference in different genders in the TCGA and CGGA databases. ^NS^p>0.05. (**C**, **G**) Age showed no significant difference in recurrence risk groups in TCGA database. NS means p>0.05. Patients in the high risk recurrence group were older than the low risk group. ^**^p<0.01. (**D**, **H**) The mesenchymal and neural subtypes showed significant higher recurrence scores in th TCGA and CGGA databases. ^NS^p>0.05, ^**^p<0.01, ^***^p<0.001, ^****^p<0.0001. (**E**, **I**) The recurrence score showed no significant difference in different MGMT promoter status GBM samples in TCGA and CGGA databases. ^NS^p>0.05.

The recurrent risk scores and clinicopathologic characteristics of 545 GBMs are displayed in Figure 2A. As shown in the heatmap, progression-free survival time gradually shortens as recurrent risk scores increase. In addition, we also noticed that transcriptome subtype of GBM showed asymmetric distribution characteristics with an increase of risk score. However, gender, Age, KPS Scores, MGMT promoter status, and postoperative adjuvant therapy showed no significant correlation with risk score. The distribution of the 6 representative genes is also shown in the heatmap. Subsequently, statistical analysis of these findings was conducted.

### The recurrent risk score was closely related to the molecular subtype of GBM

There was no close correlation between the recurrent risk score and clinical features, such as gender and age, of GBM (Figure 2B and 2C). The transcriptome subtype is one of the most important molecular features of GBM. As shown in Figure 2D, GBM classified as the mesenchymal subtype have significantly higher recurrent risk scores. MGMT promoter status was an important indicator of the chemotherapy sensitivity of GBM. There were no significant differences in recurrent risk scores between GBMs with different MGMT promoter statuses (Figure 2E). Importantly, the above results were verified independently in CGGA database (Figure 2F–2I).

### The recurrent risk score reflected the proportion of lymphocytes in GBM

**Figure 3:**
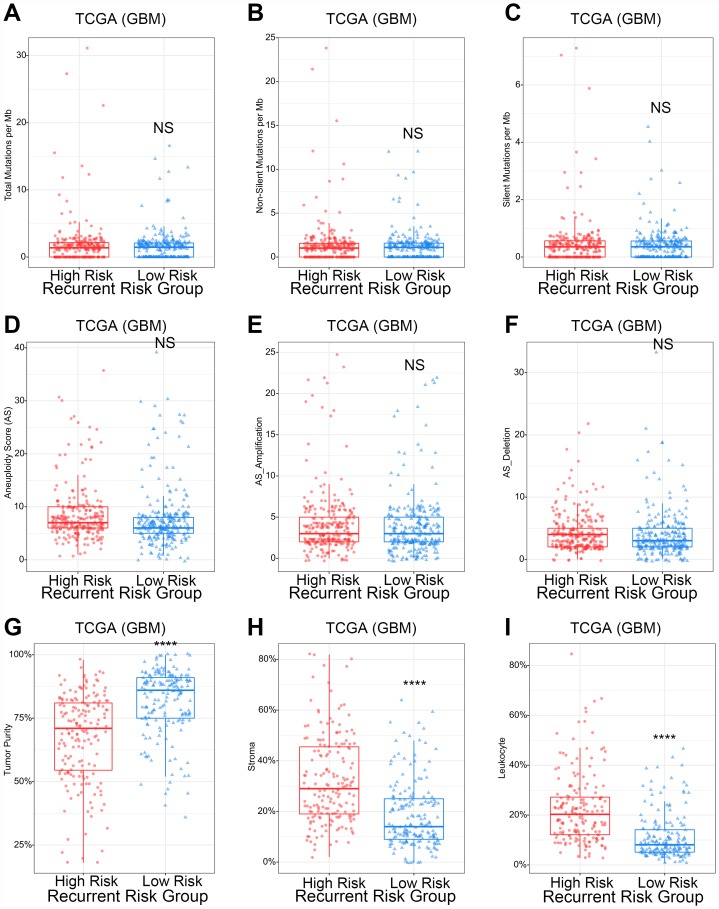
**The relationship between recurrence score and gene mutation, aneuploidy scores, and tumor purity of GBM.** (**A**–**C**) The gene mutation counts showed no significant difference in different recurrence risk groups. ^NS^p>0.05. (**D**–**F**) The aneuploidy score showed no significant difference in different recurrence risk groups. ^NS^p>0.05. (**G**–**I**) High recurrence risk group with lower tumor purity, higher stroma proportion and higher leukocyte proportion. ^****^p<0.0001.

**Figure 4:**
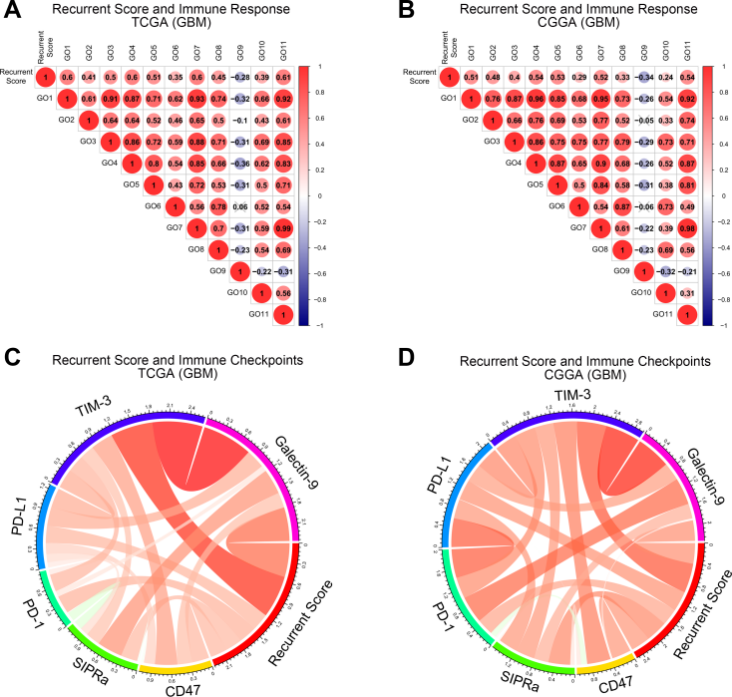
**The relationship between recurrence score and tumor immune functions of GBM.** (**A**, **B**) Correlation matrix of recurrence score and tumor immune functions in the TCGA and CGGA databases. The numbers were the R value of the Pearson correlation analysis. GO1: Immune response. GO2: B cell activation involved in immune response. GO3: T cell activation involved in immune response. GO4: Cytokine production involved in immune response. GO5: Cytokine secretion involved in immune response. GO6: Immune response to tumor cell. GO7: Leukocyte activation involved in immune response. GO8: Natural killer cell-mediated immune response to tumor cell. GO9: T cell-mediated immune response to tumor cell. GO10: Natural killer cell activation involved in immune response. GO11: Myeloid cell activation involved in immune response. (**C**, **D**) Correlation matrix of recurrence score and immune checkpoints in TCGA and CGGA databases. Red strips represent positive correlations and green strips represent negative correlations. The wider the strip, the stronger the correlation. The p-value of the correlation analysis between recurrence score and tumor immune functions was provided in Supplementary Table 2.

Subsequently, in-depth analysis of the recurrent risk score in GBM was performed. At the DNA level, there was no significant correlation between the risk score and gene mutations, including total mutations, non-silent mutations or silent mutations (Figure 3A–3C). At the chromosome level, the risk score was also not related to changes in chromosomes, including chromosome amplification or deletion (Figure 3D–3F). Tumor purity, a concept that has received wide attention in recent years, has important biological significance in GBM. Our results showed that the higher the risk score, the lower the tumor purity (Figure 3G and 3H). Further studies found that the risk score was closely related to the proportion of leukocytes in the stroma of GBM (Figure 4I). The above results indicated that the risk score may be closely related to tumor immunity. To verify this speculation, further analysis was conducted.

### Patients with higher risk scores suffered from stronger tumor immunosuppression

To explore the relationship between the risk score and immune functions, a correlation analysis between the risk score and immune process scores was performed. The risk score was significantly positively correlated with almost all immune functions, but significantly negatively correlated with T cell-mediated immune responses to tumor cells in both the TCGA and CGGA databases (Figure 4A and 4B). This result made us think of the immunosuppressive function of the immune checkpoint pathways. Interestingly, the risk scores were significantly positively correlated with the expression of immunological checkpoint receptors and ligands in the CGGA and TCGA databases (Figure 4C and 4D).

### The quantitative risk assessment system could predict the probability of postoperative recurrence of GBM at various time points

**Figure 5:**
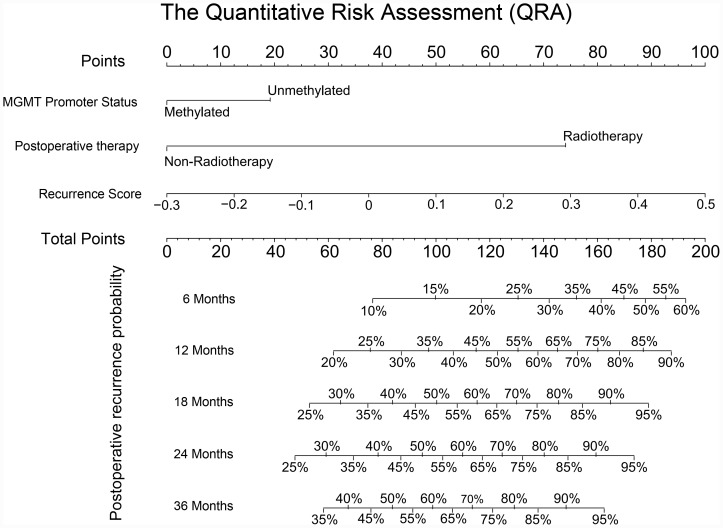
**The quantitative risk assessment for PFS prediction in GBM.** The nomogram was the quantitative risk assessment for PFS prediction in GBM. The upper part is the scoring system and the lower part is the prediction system. The probability of recurrence time of each GBM patient after surgery could be predicted according to total points.

To facilitate the clinical transformation of these results, a fracture map of the quantitative risk assessment system was constructed. As shown in Figure 5, the fracture map was divided into two parts. The upper part was used for point scoring and the lower part was used for the resulting prediction. First, a GBM patient was scored based on his or her MGMT promoter status, postoperative therapy, and recurrence score. Then, clinicians could predict the recurrence probability for that patient over 6 months, 12 months, 18 months, 24 months, and 36 months after the intracranial tumor resection. Importantly, the quantitative risk assessment system showed high prediction accuracy in both TCGA and CGGA databases (Supplementary Figure 2).

### The quantitative risk assessment system could predict overall survival and progression-free survival of GBM in retrospective analysis

**Figure 6:**
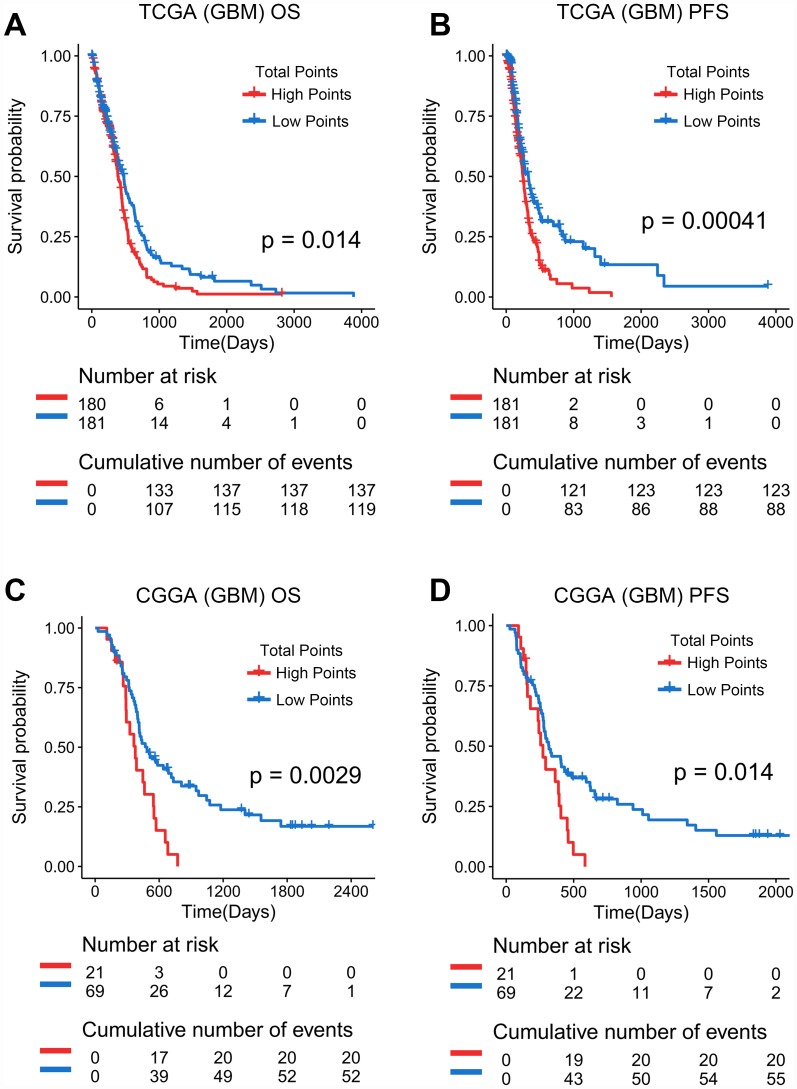
**Prediction effect of the quantitative risk assessment in GBM.** (**A**, **B**) Kaplan-Meier Curves showed that GBM patients classified in the high points group have a shorter overall survival and progression-free survival in TCGA database. (**C**, **D**) Kaplan-Meier Curves showed that GBM patients classified in the high points group have a shorter overall survival and progression-free survival in CGGA database.

In a retrospective analysis, Kaplan-Meier Curves showed that GBM patients in TCGA with a higher total score on the quantitative risk assessment system have shorter overall survival and progression-free survival time (Figure 6A and 6B). The cutoff was defined as the median of the total points of all patients. In addition, the above results could be independently verified in the CGGA database (Figure 6C and 6D). The CGGA and TCGA databases used the same cutoff value. In summary, the quantitative risk assessment system not only predicted the progression free survival but also predicted overall survival in GBM patients.

### Gallic acid increases the sensitivity of glioma cells to temozolomide by ROS scavenging

**Figure 7:**
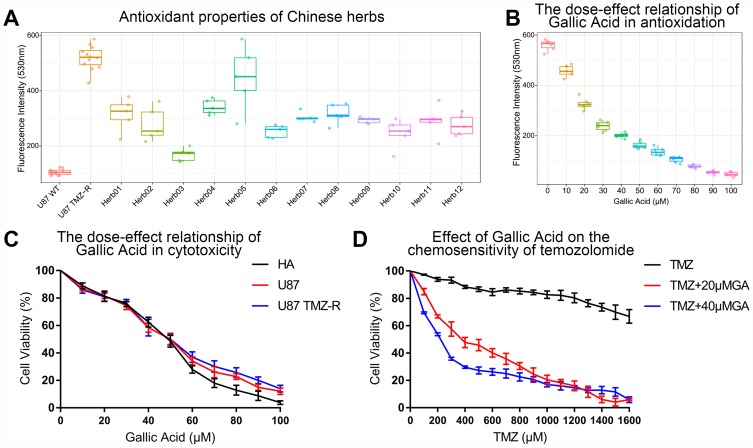
**Gallic acid was screened out as a TMZ chemotherapy sensitizer in TMZ-resistant glioma cells.** (**A**) Gallic acid showed the strongest ROS scavenging ability in 12 kinds of herbs extracts in U87 TMZ-R. Herb1: Atractylenolide I, Herb2: Curcumin, Herb3: Gallic acid, Herb4: Nuzhenide, Herb5: Pachymic acid, Herb6: Pulchinenoside C, Herb7: Paeoniflorin, Herb8: Curcumol, Herb9: Polydatin, Herb10: Danshensu, Herb11: Artemisinin, Herb12: Scutellarin. (**B**) The ROS scavenging capacity increased with increasing concentration of gallic acid in U87 TMZ-R. (**C**) The cytotoxicity increased with increasing concentration of gallic acid in HA, U87, and U87 TMZ-R. (**D**) A low dose of gallic acid (20 μM and 40 μM) could restore chemosensitivity of U87 TMZ-R to temozolomide.

Compared with wild-type U87, we found that the ROS of TMZ-resistant U87 was significantly elevated *in vitro*. After treating TMZ-resistant U87 with 12 kinds of Chinese herbal extracts with antioxidant effects, we found that all herbs except pachymic acid can scavenge ROS of glioma cells *in vitro*. Gallic acid showed the strongest antioxidant effect in TMZ-resistant U87 (Figure 7A). Gallic acid appeared to have a dose dependent antioxidant effect (Figure 7B). In addition, we found that gallic acid has non-selective cytotoxicity, which was also dose-dependent (Figure 7C). The tumor cell-killing effect of TMZ was significantly enhanced by the combination of low-dose gallic acid and TMZ (Figure 7D).

## DISCUSSION

GBM, as a highly heterogeneous tumor, is resistant to traditional therapy and is often highly recurrent [19–21]. However, with the rapid development of sequencing technology in the past years, a large number of drug resistance molecular markers have been discovered [22–24]. Based on these new targets, the sensitivity of GBM to chemotherapeutic drugs can be predicted. For example, the sensitivity of GBM to temozolomide can be predicted by the methylation level of the MGMT promoter region [25, 26]. In addition, numerous molecular targeted drugs have also been developed due to the discovery of new targets [23, 27, 28]. However, these drugs usually benefit only a small number of patients due to the heterogeneity of tumors among patients. Therefore, therapeutic targets with wide applicability need to be discovered. Unlike many previous studies, biological functions of GBM were the basis of our research in this study. After a series of comparative analyses, we found that ROS biosynthetic processes were significantly elevated in recurrent GBM. This finding was verified in paired primary/recurrent GBM samples.

Dimensionality analysis on ROS-related genes was carried out. ROS-related genes were reduced from 23 to 6 by LASSO-COX dimensionality reduction analysis [29–31]. By detecting the expression of these 6 genes, the recurrence score for each GBM patient could be obtained. We found that the recurrence score was closely related to the molecular pathology of GBM. The recurrence score was significantly elevated in mesenchymal and neural subtypes of GBM. Meanwhile, GBM with higher recurrence score often had lower tumor purity and higher leukocyte ratios. Both of these findings suggested that the recurrence score may be associated with tumor immune functions. We assessed this hypothesis by correlation analysis between the recurrence score and tumor immune functions. Interestingly, we found that the recurrence score was positively correlated with all immune functions except T cell mediated immune responses to tumor cells. This phenomenon made us think of the functional features of immune checkpoints [32]. As expected, subsequent analysis revealed a significant positive correlation between the recurrence score and receptors and ligands of common immune checkpoints in GBM. Further analysis also found that the recurrence score has the strongest correlation with TIM-3, a novel immune checkpoint with great potential for clinical application [33–35]. The above results indicated that the recurrence score has potential clinical value in guiding the immunotherapy of GBM.

Tumor recurrence is the main cause of failure of GBM treatment. Early warnings of tumor recurrence and early intervention of recurrent tumors are important for prolonging the survival time of GBM patients. At present, regular MRI review and monitoring of patient symptoms are the most common way to determine whether a tumor has recurred. However, this method relies upon detecting a tumor that has already recurred, which can lead to an increase in the patient's medical expenses or significant delays in treatment. To solve this clinical problem, we tried many risk prediction models and found a quantitative risk assessment (QRA) system based on nomogram was the most appropriate model for prediction of tumor recurrence time. QRA has been widely used in risk prediction in many fields due to its simplicity and reliability [36, 37]. When constructing the predictive model, several known prognostic correlation factors such as age, KPS, MGMT promoter status, radiotherapy, chemotherapy, and the recurrence score were included, and finally obtained a predictive model based on three predictors. The probability of recurrence of GBM at various time points after surgery could be predicted by this QRA system. The prediction system could guide the establishment of individualized review programs for postoperative GBM patients and improve the effective utilization of medical resources.

In the treatment of recurrent GBM, we screened ROS scavengers in Chinese herbal extracts with anti-oxidant properties. Gallic acid has the strongest ROS scavenging activity and was selected as the candidate drug for further study. Unexpectedly, we found that high dose gallic acid has a strong antioxidant effect as well as cytotoxicity in both an astrocyte cell line (HA) and a GBM cell line (U87). In previous reports, gallic acid was found to inhibit the proliferation of GBM [38, 39]. However, we found that the inhibition of cell proliferation by gallic acid was not tumor-specific. Fortunately, our results showed low-dose gallic acid, with its high anti-oxidant effects and low cytotoxicity, could significantly improve the chemosensitivity of TMZ-resistant glioma cells. Fewer side effects and stronger therapeutic effects make gallic acid a potential drug for adjuvant treatment of patients with recurrent GBM.

In conclusion, this study found that ROS biosynthetic processes were significantly increased in recurrent GBM. A QRA system was constructed for prediction of recurrence time. Gallic acid, a ROS scavenger, was identified as a potential drug for the treatment of recurrent GBM.

## METHODS

### Sample and data collection

A total of 663 primary and recurrent GBM samples were included in this study. In the CGGA database, the transcriptome microarray data of 118 GBM samples were collected. The clinical information and molecular pathology information of each patient were downloaded from the portal sites of CGGA (http://www.cgga.org.cn/). In the TCGA database, transcriptome microarray data of 545 GBM samples and corresponding clinical information were obtained from the portal sites of TCGA (https://cancergenome.nih.gov). Clinical information of patients was provided as Table 1. Transcriptome microarray data of primary/recurrent paired GBM samples were obtained from GSE62153.

**Table 1A:** Clinical information of patients in CGGA.

**Characteristics (CGGA)**	**No. of Patients (CGGA)**
***Hisopathology diagnosis***	
Primary Glioblastoma	109
Recurrent Glioblastoma	9
***Gender***	
Female	47
Male	71
***Age at diagnosis***	
<65	112
≥65	6
***MGMG Status***	
Methylation	45
Unmethylation	55
Not Available	18
***TCGA Subtypes***	
Proneural	14
Neural	7
Classical	19
Mesenchymal	63
Not Available	15
***Postoperative Therapy***	
Radiotherapy+TMZ Chemotherapy	63
Radiotherapy	33
TMZ Chemotherapy	6
Others	16

**Table 1B:** Clinical information of patients in TCGA.

**Characteristics (TCGA)**	**No. of Patients (TCGA)**
***Hisopathology diagnosis***	
Primary Glioblastoma	529
Recurrent Glioblastoma	16
***Gender***	
Female	213
Male	332
***Age at diagnosis***	
<65	360
≥65	185
***Preoperative KPS score***	
≥80	297
<80	101
Not Available	147
***TCGA Subtypes***	
Proneural	99
Neural	85
Classical	152
Mesenchymal	161
G-CIMP	35
Not Available	13
***MGMG Status***	
Methylation	163
Unmethylation	199
Not Available	183
***Postoperative Therapy***	
Radiotherapy+TMZ Chemotherapy	358
Radiotherapy	159
TMZ Chemotherapy	11
Others	17

### Functional enrichment analysis

The functional enrichment method used in this study was Gene Set Variation Analysis (GSVA). The analysis process was performed by *gsva* package in R under the default parameters. The p-value of the correlation analysis between recurrence score and tumor immune functions was provided in Supplementary Table 2. The gene list of biological processes was downloaded from AmiGO 2 Web portals (http://amigo.geneontology.org/amigo/landing).

### Receiver Operating Characteristic (ROC) analysis

The ROC analysis was performed using the *pROC* package in R under the default parameters. The specificity of functional enrichment was represented by AUC value and p value. Drawing of ROC results of CGGA and TCGA was performed by the *ggplot2* package in R.

### Construction of the recurrent risk score

Firstly, LASSO-COX analysis was performed by *glmnet* and *survival* package in R. Lambda value of each ROS related gene was obtained by dimensionality analysis based on progression-free survival of TCGA GBM patients. The recurrent risk score was the sum of the product of gene expression and their corresponding lambda value As follows:

Risk score = expr_gene1_ × λ_gene1_ + expr_gene2_ × λ_gene2_ + … + expr_genen_ × λ_genen_

expr_gene_: expression values of genes. λ_gene_: corresponding lambda values of genes. The prognostic value of ROS related genes was provided as Supplementary Table 1.

### Construction of the quantitative risk assessment (QRA) system

The QRA system was built based on nomogram. The nomogram was performed by *rms* package in R. The age, KPS, MGMT promoter status, radiotherapy, chemotherapy, and the recurrence score were included as risk factors for recurrence of GBM. Validation analysis of prediction accuracy used default parameters.

### Cell culture

GBM cell line U87 and astrocyte cell line HA were obtained from the Institute of Biochemistry and Cell Biology, Chinese Academy of Science. U87 was cultured in the culture medium containing DMEM (Gibco) supplemented with 10% Fetal Bovine Serum (Gibco) and HA was cultured in Astrocyte Medium (Gibco). TMZ-resistant U87 (U87 TMZ-R) was derived from U87 by culturing in serum-containing medium with gradually increased temozolomide (Sigma Aldrich) to the final concentration of 1,000 μM. U87 TMZ-R was enzymatically dissociated into single cells using Trypsin/EDTA Solution (Gibco) and cultured in the serum and TMZ-containing medium every 4–6 days. Cell passage of U87 and HA were performed every 3–4 days.

### Antioxidant capacity test of Chinese herbs

Antioxidant capacity test of Chinese herbs was assessed by ROS assay kit (C1300, applygen). The experimental process was carried out strictly in accordance with the instructions of the kit. The final concentration of DCFH-DA was 10 μM. The cells were incubated at 37 °C for 60 minutes after the addition of the reaction reagent. The excitation wavelength and the emission wavelength of the multiscan spectrum (M200 Pro, Tecan) were set to 500 nm and 525 nm respectively for results detecting. Extracts of herbs were purchased from reagent company (Details in Supplementary Table 3). The test results of U87 WT and U87 TMZ-R were used as a negative control and positive control in this study. ROS detection was performed 3 hours after the addition of 50 μM of every herb in U87 TMZ-R. The lower the fluorescence intensity, the stronger the antioxidant capacity of the herb.

### Cell viability test

Cell viability of cell lines was assessed by Cell Counting Kit-8 (Dojindo). The experimental process was carried out strictly in accordance with the instructions of the kit. First, 1*10^5^ cells were cultured in 96-well plates 12–24 hours before the experimental intervention. Cell viability testing was performed after 72 hours of exposure to experimental drugs. The cells were incubated at 37 °C for 120 minutes after the addition of the reaction reagent. Absorbance detection of each test was performed by multiscan spectrum (M200 Pro, Tecan). Four parallel controls were set up for every test.

### Statistical analysis

Statistical analyses and drawings were performed by R (https://www.r-project.org/, v3.5.0), GraphPad Prism software and Microsoft office 365. Lambda values of ROS related genes were shown by GraphPad Prism and the clinical pathological characteristics were drawn by Microsoft Office 365. Other statistical computations and figures were generated by several R packages. A p value less than 0.05 was considered statistically significant. All statistical tests were two-sided.

## Supplementary Material

Supplementary Figures

Supplementary Tables
