# Supplementary material for: Reactive oxygen species metabolism-based prediction model and drug for patients with recurrent glioblastoma
Source: Aging (Albany NY). 2019 Dec 4;11(23):11010–29. doi: 10.18632/aging.102506 (PMC6932921; doi:10.18632/aging.102506)
Supplement: Supplementary Figures [file aging-11-102506-s002..pdf]

SUPPLEMENTARY FIGURES

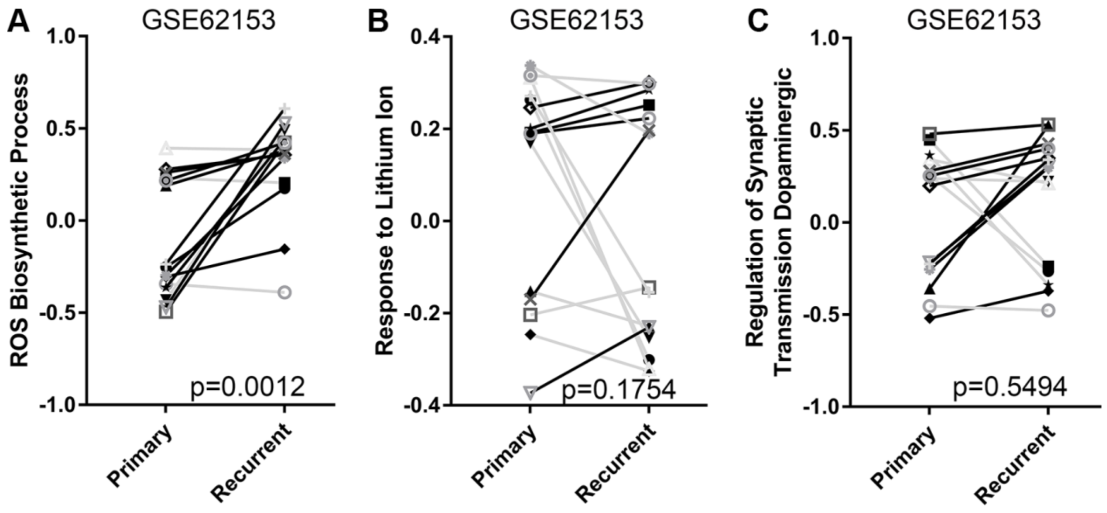

Supplementary Figure 1. Reactive oxygen species biosynthetic process was significantly elevated in recurrent GBM compared to paired primary one. (**A**) ROS biosynthetic process scores significantly increased in recurrent tumors. (**B** and **C**) Scores of response to lithium ion and regulation of synaptic dopaminergic transmission showed no significant change with tumor recurrence. Black lines represent elevation and gray lines represent decline of functional scores.

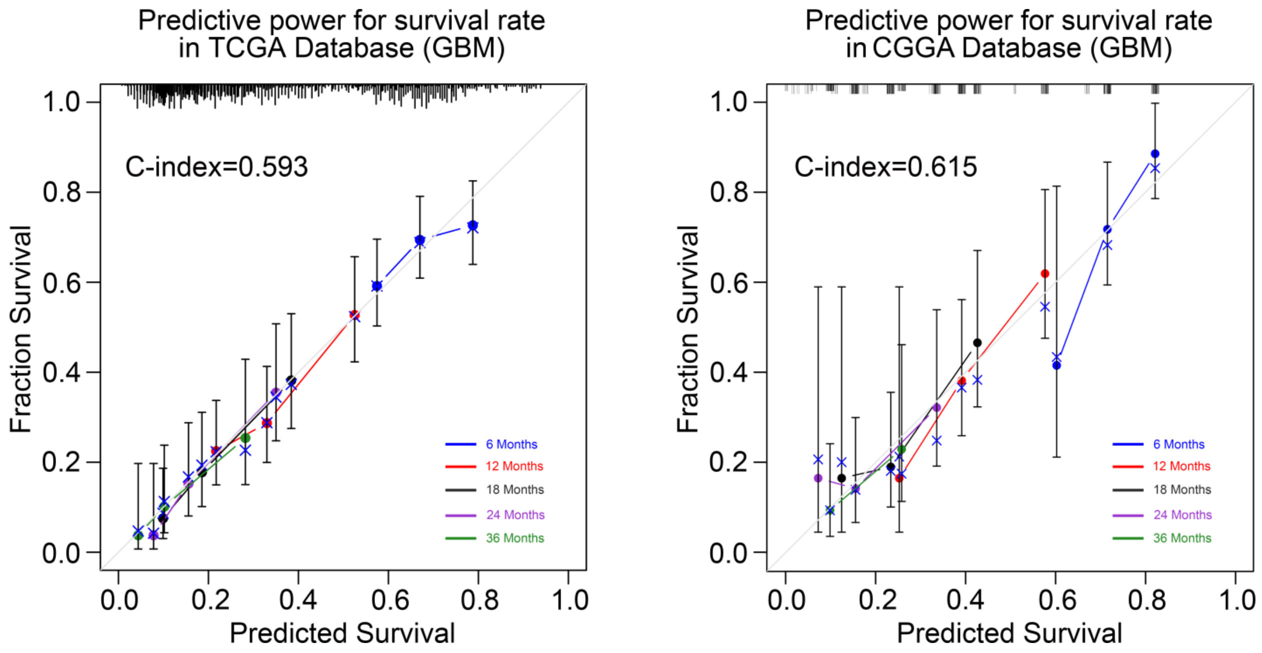

Supplementary Figure 2. Predictive power of the quantitative risk assessment in GBM. The quantitative risk assessment showed good predictive effects on the probability of recurrence in 6, 12, 18, 24, and 36 months after surgery in the TCGA and CGGA databases.
