# Supplementary material for: Reactive oxygen species metabolism-based prediction model and drug for patients with recurrent glioblastoma
Source: Aging (Albany NY). 2019 Dec 4;11(23):11010–29. doi: 10.18632/aging.102506 (PMC6932921; doi:10.18632/aging.102506)
Supplement: Supplementary Tables [file aging-11-102506-s001..pdf]

SUPPLEMENTARY TABLES

Supplementary Table 1A. The prognostic value of ROS related genes in CGGA GBM OS.

Gene	HR	95% CI		p-value
		lower	upper	
AKT1	1.118	0.754	1.657	0.579
ARG2	1.122	0.842	1.497	0.432
CYBA	1.158	0.801	1.676	0.435
CYBB	1.001	0.723	1.386	0.995
CYP1A1	0.740	0.515	1.063	0.103
CYP1A2	0.914	0.679	1.230	0.553
CYP1B1	1.061	0.951	1.182	0.289
DDAH2	1.026	0.695	1.515	0.896
DUOX1	0.970	0.806	1.167	0.747
DUOX2	0.980	0.752	1.276	0.879
GBF1	1.031	0.792	1.343	0.819
GCH1	1.316	1.009	1.716	0.043
GCHFR	1.536	1.194	1.975	0.001
MAOB	1.151	0.995	1.330	0.058
MPO	1.146	0.918	1.431	0.230
NOS1	0.991	0.742	1.326	0.954
NOS3	1.142	0.786	1.660	0.486
NQO1	1.044	0.822	1.326	0.724
RORA	0.978	0.626	1.529	0.923
SLC7A2	0.969	0.729	1.287	0.826
SOD1	0.925	0.639	1.338	0.678
SPR	1.235	0.856	1.783	0.259

Supplementary Table 1B. The prognostic value of ROS related genes in CGGA GBM PFS.

Gene	HR	95% CI		p-value
		lower	upper	
AKT1	1.177	0.804	1.724	0.402
ARG2	1.078	0.819	1.419	0.592
CYBA	1.016	0.716	1.442	0.928
CYBB	0.976	0.705	1.350	0.883
CYP1A1	0.776	0.557	1.082	0.135
CYP1A2	0.876	0.652	1.177	0.379
CYP1B1	1.039	0.934	1.157	0.479
DDAH2	1.158	0.783	1.714	0.462
DUOX1	0.933	0.777	1.119	0.452
DUOX2	0.943	0.726	1.225	0.660
GBF1	0.993	0.758	1.301	0.960
GCH1	1.194	0.916	1.557	0.189
GCHFR	1.345	1.062	1.702	0.014
MAOB	1.119	0.973	1.287	0.116
MPO	1.118	0.905	1.381	0.299
NOS1	1.013	0.762	1.348	0.929
NOS3	1.139	0.787	1.649	0.490
NQO1	0.974	0.774	1.226	0.824
RORA	0.959	0.625	1.473	0.849
SLC7A2	0.901	0.687	1.183	0.453
SOD1	0.865	0.614	1.218	0.405
SPR	1.098	0.758	1.589	0.621

Supplementary Table 1C. The prognostic value of ROS related genes in TCGA GBM OS

Gene	HR	95% CI		p-value
		lower	upper	
AKT1	1.119	0.991	1.262	0.069
ARG2	0.985	0.877	1.107	0.802
CYBA	1.009	0.890	1.145	0.888
CYBB	1.087	0.987	1.198	0.091
CYP1A1	0.813	0.696	0.950	0.009
CYP1A2	0.800	0.564	1.134	0.209
CYP1B1	1.007	0.953	1.063	0.815
DDAH2	0.981	0.820	1.174	0.836
DUOX1	0.990	0.864	1.134	0.883
DUOX2	0.654	0.451	0.947	0.025
GBF1	0.933	0.762	1.143	0.505
GCH1	0.942	0.809	1.098	0.444
GCHFR	1.107	0.973	1.260	0.123
MAOB	1.112	1.039	1.190	0.002
MPO	1.176	1.021	1.355	0.024
NOS1	1.017	0.633	1.632	0.946
NOS3	0.729	0.592	0.898	0.003
NQO1	0.994	0.885	1.115	0.913
RORA	0.989	0.896	1.092	0.826
SLC7A2	0.966	0.835	1.119	0.646
SOD1	0.947	0.787	1.138	0.558
SPR	1.096	0.951	1.264	0.206

Supplementary Table 1D. The prognostic value of ROS related genes in TCGA GBM PFS.

Gene	HR	95% CI		p-value
		lower	upper	
AKT1	1.016	0.888	1.161	0.822
ARG2	1.094	0.966	1.239	0.155
CYBA	1.006	0.878	1.152	0.933
CYBB	1.126	1.014	1.251	0.027
CYP1A1	0.928	0.778	1.106	0.403
CYP1A2	0.742	0.512	1.074	0.113
CYP1B1	1.045	0.983	1.111	0.158
DDAH2	0.828	0.676	1.015	0.069
DUOX1	0.900	0.774	1.046	0.170
DUOX2	0.808	0.536	1.216	0.307
GBF1	0.959	0.773	1.191	0.706
GCH1	1.168	0.997	1.368	0.054
GCHFR	1.025	0.885	1.187	0.742
MAOB	1.119	1.039	1.205	0.003
MPO	1.114	0.948	1.308	0.190
NOS1	0.890	0.528	1.502	0.663
NOS3	0.864	0.689	1.085	0.208
NQO1	1.153	1.016	1.308	0.027
RORA	0.991	0.888	1.107	0.876
SLC7A2	0.993	0.849	1.162	0.931
SOD1	1.071	0.871	1.317	0.513
SPR	1.075	0.920	1.258	0.363

Supplementary Table 2A. Recurremt score and immune response (TCGA GBM).

Terms	R-value (Pearson)	95% confidence interval	P-value
Immune response	0.5973	0.5392 to 0.6498	<0.0001
B cell activation involved in immune response	0.4144	0.3408 to 0.483	<0.0001
T cell activation involved in immune response	0.5039	0.437 to 0.5652	<0.0001
Cytokine production involved in immune response	0.5977	0.5396 to 0.6501	<0.0001
Cytokine secretion involved in immune response	0.5105	0.4442 to 0.5713	<0.0001
Immune response to tumor cell	0.3471	0.2694 to 0.4204	<0.0001
Leukocyte activation involved in immune response	0.6013	0.5435 to 0.6533	<0.0001
Natural killer cell mediated immune response to tumor cell	0.4526	0.3817 to 0.5183	<0.0001
T cell mediated immune response to tumor cell	-0.2789	-0.3561 to -0.1979	<0.0001
Natural killer cell activation involved in immune response	0.3899	0.3147 to 0.4603	<0.0001
Myeloid cell activation involved in immune response	0.6093	0.5524 to 0.6605	<0.0001

Supplementary Table 2B. Recurremt score and immune response (CGGA GBM).

Terms	R-value(Pearson)	95% confidence interval	P-value
Immune response	0.513	0.3662 to 0.6349	<0.0001
B cell activation involved in immune response	0.476	0.323 to 0.6047	<0.0001
T cell activation involved in immune response	0.4002	0.2365 to 0.5417	<0.0001
Cytokine production involved in immune response	0.5401	0.3982 to 0.6567	<0.0001
Cytokine secretion involved in immune response	0.533	0.3898 to 0.6511	<0.0001
Immune response to tumor cell	0.2925	0.1179 to 0.4495	0.0013
Leukocyte activation involved in immune response	0.5174	0.3713 to 0.6384	<0.0001
Natural killer cell mediated immune response to tumor cell	0.331	0.1598 to 0.4828	0.0003
T cell mediated immune response to tumor cell	-0.3358	-0.487 to -0.1651	0.0002
Natural killer cell activation involved in immune response	0.2384	0.06027 to 0.4019	0.0093
Myeloid cell activation involved in immune response	0.5352	0.3924 to 0.6528	<0.0001

Supplementary Table 2C. Recurremt score and immune checkpoints.

Immune Checkpoints	Gene	CGGA GBM		TCGA GBM	
		R-value (Pearson)	P-value	R-value (Pearson)	P-value
CD47	CD47	0.381281364	2.05E-05	0.26475689	3.41E-10
SIPR α	SIRPA	0.430000493	1.18E-06	0.262529541	4.84E-10
PD-1	PDCD1	0.329448749	0.000269607	0.285301926	1.15E-11
PD-L1	CD274	0.399617189	7.36E-06	0.270888019	1.28E-10
TIM-3	HAVCR2	0.583241346	4.20E-12	0.669030442	5.36E-72
Galectin-9	LGALS9	0.407260225	4.72E-06	0.533192414	2.32E-41

Supplementary Table 3.

ID	Herbs (English)	Herbs (Chinese)	Extract of Herbs (English)	Extract of Herbs (Chinese)	Drug Company	Item No
Herb1	Atractylodes Macrocephala Koidz	白术	Atractylenolide I	白术内酯	MCE	HY-N0201
Herb2	Curcuma longa	姜黄	Curcumin	姜黄素	Selleck	S7501
Herb3	Nutgall	五倍子	Gallic acid	没食子酸	MCE	HY-N0523
Herb4	Glossy Privet Fruit	女贞子	Nuzhenide	女贞子甙	Selleck	S9473
Herb5	Poria	茯苓	Pachymic acid	茯苓酸	MCE	HY-N0371
Herb6	Pulsatilla Chinensis	白头翁	Pulchinenoside C	白头翁皂苷 C	MCE	HY-N0205
Herb7	Radix Paeoniae Alba	白芍	Paeoniflorin	芍药苷	MCE	HY-N0293
Herb8	Rhizoma Curcumae	莪术	Curcumol	莪术醇	MCE	HY-N0104
Herb9	Rhizoma Polygoni Cuspidati	虎杖	Polydatin	虎杖苷	MCE	HY-N0120A
Herb10	Salviamiltiorrhiza	丹参	Danshensu	丹参素	MCE	HY-N1913
Herb11	Sweet Wormwood Herb	青蒿	Artemisinin	青蒿素	MCE	HY-B0094
Herb12	Wild Skullcaps	野生黄芩	Scutellarin	野黄芩苷	MCE	HY-N0751
